# Mis-Spliced Transcripts of Nicotinic Acetylcholine Receptor α6 Are Associated with Field Evolved Spinosad Resistance in *Plutella xylostella* (L.)

**DOI:** 10.1371/journal.pgen.1000802

**Published:** 2010-01-08

**Authors:** Simon W. Baxter, Mao Chen, Anna Dawson, Jian-Zhou Zhao, Heiko Vogel, Anthony M. Shelton, David G. Heckel, Chris D. Jiggins

**Affiliations:** 1 Department of Zoology, University of Cambridge, Cambridge, United Kingdom; 2 Department of Entomology, Cornell University/New York State Agricultural Experiment Station, Ithaca, New York, United States of America; 3 Department of Entomology, Max Planck Institute for Chemical Ecology, Jena, Germany; Princeton University, Howard Hughes Medical Institute, United States of America

## Abstract

The evolution of insecticide resistance is a global constraint to agricultural production. Spinosad is a new, low-environmental-risk insecticide that primarily targets nicotinic acetylcholine receptors (nAChR) and is effective against a wide range of pest species. However, after only a few years of application, field evolved resistance emerged in the diamondback moth, *Plutella xylostella*, an important pest of brassica crops worldwide. Spinosad resistance in a Hawaiian population results from a single incompletely recessive and autosomal gene, and here we use AFLP linkage mapping to identify the chromosome controlling resistance in a backcross family. Recombinational mapping with more than 700 backcross progeny positioned a putative spinosad target, nAChR alpha 6 (*Pxα6*), at the resistance locus, *PxSpinR*. A mutation within the ninth intron splice junction of *Pxα6* results in mis-splicing of transcripts, which produce a predicted protein truncated between the third and fourth transmembrane domains. Additional resistance-associated Pxα6 transcripts that excluded the mutation containing exon were detected, and these were also predicted to produce truncated proteins. Identification of the locus of resistance in this important crop pest will facilitate field monitoring of the spread of resistance and offer insights into the genetic basis of spinosad resistance in other species.

## Author Summary

Evolving resistance to control agents, such as antibiotics or insecticides, can have major costs to human health or agricultural food production. Once a genetic mechanism for resistance to a particular compound has been identified, other resistant species can be rapidly assessed to search for a parallel mechanism. Insecticides often target the insect nervous system as they can be toxic at low concentration and act rapidly. Here we report a genetic mutation in a global agricultural pest, diamondback moth, that is associated with resistance to the bioinsecticide spinosad. A mutation in an intron splice junction of *nicotinic acetylcholine receptor* (*nAChR*) *alpha 6* causes mis-spliced mRNA transcripts that are predicted to produce truncated proteins lacking important functional domains. nAChRs require 5 subunits to function, and insects generally encode 10–12 subunit genes. Spinosad may therefore be targeting a redundant nAChR subunit not essential for survival in diamondback moth. Other insects that evolve field resistance to spinosad can now be tested to determine whether the same resistance mechanism is involved.

## Introduction

Insecticide resistance has become one of the major driving forces altering the development of integrated pest management programs worldwide. The diamondback moth, *Plutella xylostella*, is a global agricultural pest of crucifers and commonly develops resistance to insecticides in the field [1]. Resistance, defined as a change in response to selection by toxicants [2], has been reported to a wide range of chemicals with different modes of action, including pyrethroids, carbamates and organophosphates [3] as well as biologically derived insecticides Bt [4] and spinosad [5]. Understanding the mode of action of insecticides, and identifying the genetic mechanisms and mutations that confer resistance, will ultimately enable early detection of resistance alleles in the field and improve management strategies.

Resistance to spinosad emerged in field populations of *P. xylostella* at a remarkably rapid rate. For example, after only ≈2.5 years of commercial application of spinosad in Hawaii, six of 12 field collected populations were highly resistant, with toxicity ratios of >100 relative to a susceptible control strain [5]. Spinosad resistance in diamondback moth has subsequently been reported in additional populations in the USA, Thailand and Malaysia [5]–[7]. Resistance to spinosad has also been selected in laboratory strains of *Heliothis virescens* [8], *Musca domestica*, [9] and *Bactrocera dorsalis* [10] and reported in western flower thrips, *Frankliniella occidentalis*, collected from greenhouses [11].

Since its introduction in 1997, spinosad has been approved in more than 30 countries for use on over 150 different crops [12]. The insecticide targets a range of lepidopteran and dipteran pests [13], yet is relatively safe to non-target organisms [14],[15]. The active ingredients of spinosad are macrocyclic lactones, spinosyn A (primary component) and spinosyn D, produced by the actinomycete *Saccharopolyspora spinosa* [16] during fermentation [17],[18]. Upon spinosad exposure, insects experience tremors and paralysis caused by neuromuscular fatigue as the insecticide interferes with the central nervous system, which ultimately leads to death [19].

Spinosad primarily targets the nicotinic acetylcholine receptor (nAChR) [20], which plays an essential role in excitatory synaptic transmissions of insect nervous systems [21],[22]. nAChRs consist of five subunits, with extracellular N-terminal domains that bind acetylcholine, and four transmembrane domains. Five insect genomes have been mined for nAChRs, with 12 identified from *Tribolium castaneum* [23] and *Bombyx mori* [24], 11 from *Apis mellifera* [25] and 10 from both *Drosophila melanogaster* [26] and *Anopheles gambiae* [27]. Although insects generally have fewer nAChRs than vertebrates, increased subunit diversity has been reported through alternate exon splicing, exon exclusion or A-to-I pre-mRNA editing. For example, it has been estimated nAChR *Dα6* of *D. melanogaster* is theoretically capable of producing >30,000 different subunit variants [28] and there are at least 18 reported transcripts (8 of which include premature stop codons) in *T. castaneum Tcasα6* [29].

It has already been demonstrated that a nAChR *Dα6* deficiency strain of *D. melanogaster* with one chromosome carrying a deletion of *Dα6* shows 1181 fold resistance to spinosad [30]. One of the breakpoints in the opposite balancer chromosome CyO occurs within an exon of *Dα6*, fusing it to another gene. Although this prematurely truncates the coding sequence, it confers resistance without being lethal, making this gene a prime candidate for field based resistance in insect pests. However, Gao *et al.* (2007) found no significant differences in sequence or expression of the *Musca domestica* orthologue, *Mdα6* in a laboratory selected resistant strain (rspin) [31]. We have focused on field-based resistance to spinosad in a *Plutella xylostella* strain originally collected from Pearl City, Hawaii. Following further laboratory selection, resistance in the Pearl-Sel strain was shown to be a recessive and inherited as a single autosomal locus, and not due to metabolically mediated detoxification [5]. Crossing experiments have recently shown the same field evolved spinosad resistance mechanism is shared among populations isolated from Hawaii, California and Georgia [32]. Here we take a genetic linkage mapping approach to identify the chromosome carrying a field derived spinosad resistance mechanism. The *nAChR Dα6* orthologue, *Pxα6*, was mapped to the resistance locus *PxSpinR* by recombinational mapping, and a mutation in the 5′ donor site of intron 9 was found to cause mRNA mis-splicing thereby introducing an additional 40 bases into the mRNA of the resistant strain. This mutation leads to a premature termination codon between transmembrane domains 3 and 4 and is the likely functional cause of resistance. Further analysis around this gene region revealed complex transcript splice patterns that result in multiple frame shift mutations in the resistant, but not susceptible strain.

## Results

### Linkage group 1 contains the spinosad resistance locus, *PxSpinR*

Spinosad resistance in *Plutella xylostella* was predicted to be caused by a single, autosomal recessive gene [5]. We used biphasic linkage analysis, as previously employed in mapping Bt-resistance in *P. xylostella* [33], to identify the chromosome and localized region containing the resistance gene. Crosses were prepared between a spinosad susceptible Geneva 88 female and a spinosad resistant BCS3-Pearl male. Some F_1_ progeny were bio-assayed with a diagnostic dose of spinosad (10ppm), with no survival, demonstrating that resistance is recessive at this dosage. Single pair “female informative” backcrosses were established between an F_1_ female and a BCS3-Pearl male. The backcross progeny were expected to segregate 1∶1 for spinosad resistance or susceptibility. Approximately 70 sibling larvae were treated with 10 ppm spinosad to kill any heterozygous susceptible progeny, leaving 35 “bioassay survivors”, while 32 “untreated controls” were not exposed to insecticide. Bioassay survivors and untreated controls were reared to adults, and genomic DNA isolated for molecular analysis.

**Figure 1:**
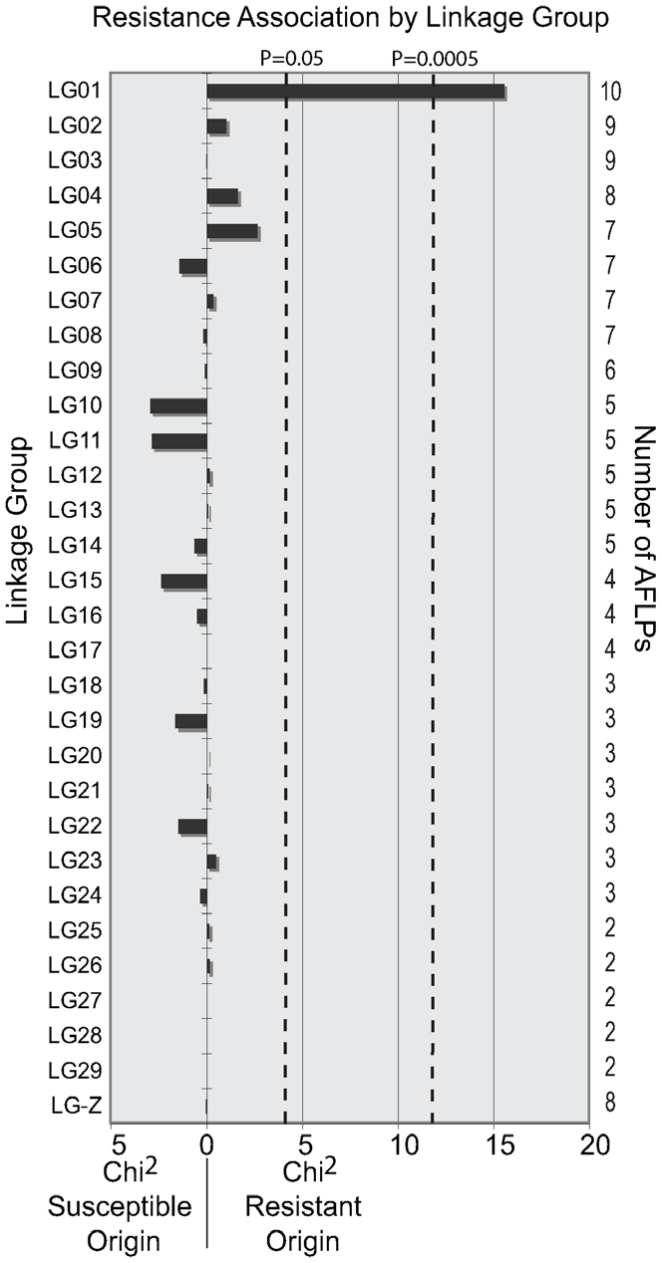
Spinosad resistance is associated with a single linkage group in *Plutella xylostella*. 146 AFLP genotypes were generated from a female informative backcross and assigned into 30 linkage groups. χ^2^ values for each linkage group were calculated by comparing genotypes inherited by backcross spinosad bioassay survivors with untreated controls. A directional bias towards spinosad susceptible or resistant grandparental origin is shown. Linkage group 1 (LG01) was significantly associated with spinosad resistance after Bonferroni correction for multiple comparisons (LG01, χ^2^ = 15.53, P>0.0001). The remaining 29 linkage groups identified here were not associated with resistance. LG-Z is the sex chromosome.

Female Lepidoptera do not undergo crossing over between chromatids during oogenesis [34]–[36]. Consequently, the chromosomes inherited from the mother are passed to the next generation as complete units. All genes and molecular markers on the same chromosome are therefore linked; and we used this property to identify the linkage group containing *PxSpinR*. AFLP genotyping was performed on a BCS3-Pearl grandfather, Geneva88 grandmother, F_1_ mother, BCS3-Pearl backcross father, 20 F_2_ untreated controls and an average of 19 F_2_ spinosad bioassay survivors. 146 variable AFLP markers inherited from the F_1_ mother were scored and assigned to 30 of the expected 31 linkage groups, each containing between 2 and 10 markers. The origin of each AFLP marker from the F_1_ mother could be associated with the resistant grandfather or susceptible grandmother. Following this, 2×2 χ^2^ tests were performed for each linkage group, comparing the number of susceptible and resistant AFLP genotypes inherited in the untreated controls with the spinosad bioassay survivors. A single linkage group was significantly associated with spinosad resistance, with all bioassay survivors inheriting the resistance derived LG01 (χ^2^ = 15.53, P>0.0001) (Figure 1).

### nAChR *Pxα6* maps to the resistance linkage group

A *P. xylostella* cDNA pool derived from egg and larval tissue was sequenced using 454-FLX sequencing technology (Roche). This provided transcriptome sequence to search for resistance candidate genes, however, *nAChR Pxα6* was not present in this dataset. Consequently, PCR with degenerate primers was used to amplify a *nAChR α6* gene fragment from larval cDNA (amino acids 105–304) with 92% identity to the *Drosophila* homologue *Dα6*. Species specific primers were designed for gene mapping, and *Pxα6* genetically mapped to the spinosad resistance linkage group, LG01. All 35 backcross progeny that survived exposure to spinosad inherited the same BCS3-Pearl derived linkage group from the F_1_ mother, while 32 untreated controls segregated 15∶17 for the susceptible or resistant derived chromosome respectively.

### Recombinational mapping positions *Pxα6* at the resistance locus

As chromosomal crossing over occurs during spermatogenesis, distances between markers on the same chromosome can be estimated based on recombination rates using the progeny of male informative crosses (F_1_ male backcrossed to a female) in the second step of biphasic linkage analysis. Male informative mapping families were generated from 31 F_1_ brothers who were backcrossed to BCS3-Pearl females in single pair matings. Bioassays with 15 ppm spinosad were performed on 2315 backcross progeny, of which 884 survived (38% survival).

**Figure 2:**
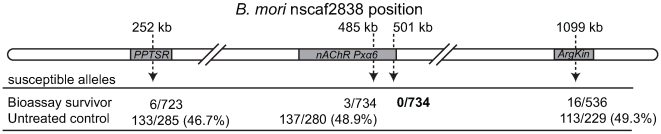
Recombinational map of the *Plutella xylostella* spinosad resistance locus, *PxSpinR*. Genes flanking *nAChR Pxα6* were chosen for genotyping based on relative position within the *B. mori* genome (distances are shown). Susceptible and resistant alleles were inherited in untreated controls at a ∼1∶1 ratio. The 3′ end of *nAChR Pxα6* was completely linked to the spinosad resistance locus.

To determine whether *nAChR Pxα6* mapped to the *PxSpinR* locus, DNA was extracted from 24 of the male informative backcrosses, totalling 734 bioassay survivors and 286 untreated controls. A genotyping assay using a polymorphism within intron 5 of *Pxα6* showed that only 3/734 bioassay-survivors inherited the allele from the spinosad susceptible strain, compared to 48.9% of controls, demonstrating that this marker was tightly linked to the spinosad resistant mutation. At any polymorphism causally responsible for resistance, however, no susceptible alleles would be expected among survivors, since F_1_ heterozygotes cannot survive the concentration of spinosad used in the bioassay. To determine whether the resistance causing mutation was up- or down-stream of *Pxα6* intron 5, candidate markers for genes flanking *Pxα6* were identified from the genome of silkmoth *Bombyx mori* and BLASTed against *P. xylostella* 454 cDNA sequences. Genotyping assays were developed for flanking genes *phosphatidylserine receptor* (PPTSR) and *arginine kinase* (ArgKin). Genotyping in PPTSR identified 6/723 recombinants, including the same three individuals from *nAChR Pxα6* intron 5, showing this was further from the resistance locus. Genotyping in *arginine kinase* had 16/536 recombinants, none of which were present at *Pxα6* intron 5. Hence the spinosad resistance region mapped between *Pxα6* intron 5 and *arginine kinase*. A second *Pxα6* PCR genotyping assay spanning intron 11 of *nAChR Pxα6* was performed on all recombinants and a subset of progeny that were nonrecombinant in this region. Here, all bioassay survivors had the same BCS3-Pearl derived resistant genotype showing complete linkage with the spinosad resistance locus, *PxSpinR* (Figure 2).

### Genomic sequencing of *Pxα6*

**Figure 3:**
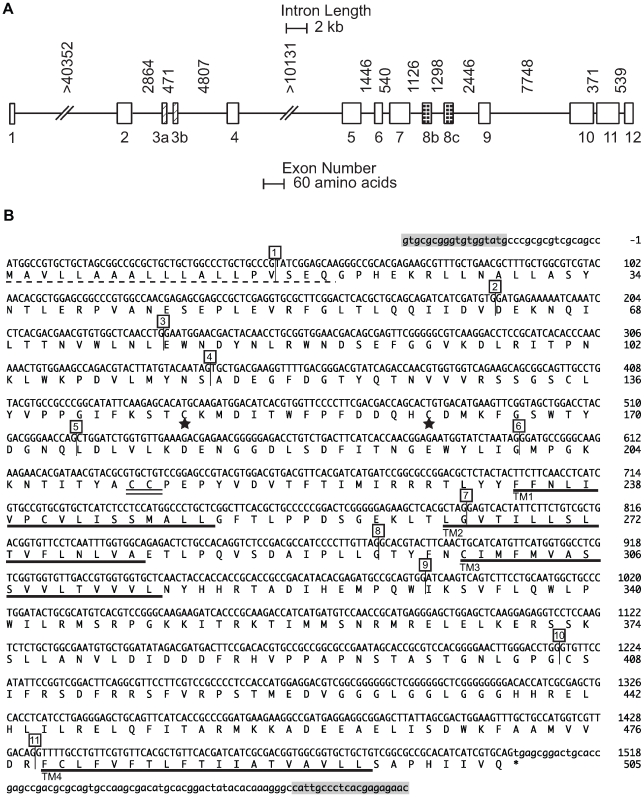
*nAChR Pxα6 g*ene and coding sequence. (A) Intron distances and relative exon sizes of *nAChR Pxα6* from Geneva 88 BAC clone Px8d14. Exon variants 3a, 3b, 8b, and 8c are shown. Scale bars differ for intron length and exon size. Introns 1 and 4 contain sequencing gaps. (B) *nAChR Pxα6* coding sequence, containing exons 3a and 8b. The predicted N-terminal signal leader peptide (probability = 0.988) is shown with a dashed line. The four transmembrane domains are underlined in bold (TM1-4), signature cysteines of nAChR alpha subunits are double underlined and neurotransmitter-gated ion-channels signature of cysteines, separated by 13 amino acids, shown with stars. Intron positions are shown in numbered boxes. PCR primers in the 5′ and 3′ UTRs (shaded boxes) amplified a product from cDNA of Geneva88 4^th^ instar larvae (GenBank GU207835).

To identify predicted coding and intragenic sequence of *Pxα6*, a *P. xylostella* genomic BAC library was constructed using susceptible strain Geneva88, 23K clones printed to nylon membrane filters, then hybridised with a cDNA amplicon covering a portion of the *Pxα6* coding sequence. Clone Px8d14 was identified, sequenced and assembled into 7 ordered fragments covering >126 kb. The predicted full length *nAChR Pxα6* coding sequence was identified, based on homology with *B. mori* (GenBank ABV45518), spanning twelve exons plus the alternative exon versions 3a, 3b, 8b and 8c reported from other insects. The full-length gene from start methionine to stop codon spanned >75 kb of the 126 kb BAC clone (GenBank GU058050, Figure 3A). To verify the coding sequence annotation, primers were designed in predicted 5′ and 3′ untranslated regions and amplified from cDNA of a 4^th^ instar Geneva88 larva using a proof reading polymerase (GenBank GU207835, Figure 3B). The predicted protein sequence of the full length product was 96%, 96% and 83% similar to nAChR α6 orthologues of *B. mori* (ABP96888), *H. virescens* (AAD32698) and *D. melanogaster* (NP_723494, isoform A) respectively.

### *Pxa6* mis-splicing occurs in the spinosad resistant strain

**Figure 4:**
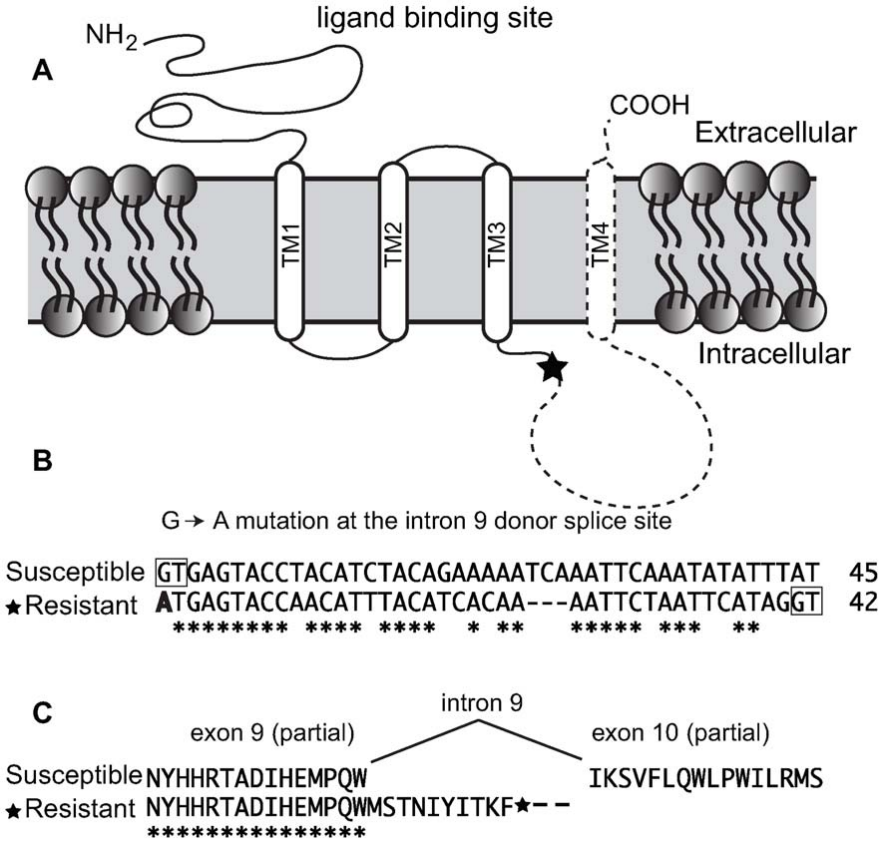
Truncating mutation of nAChR Pxα6 in spinosad resistant *Plutella xylostella*. (A) Schematic diagram of the four nAChR transmembrane domains (TM1-4). A premature stop codon in the resistant BCS3-Pearl strain is denoted with a star in the intracellular loop. The subunit region predicted to be missing is shown with dashed lines. (B) DNA sequence of intron 9 from the susceptible strain (first 45 bp), and equivalent region in the resistant strain. Intronic GT splice sites are boxed. A G→A point mutation in the resistant strain (bold) results in a mis-splicing event that introduces this 40 bp sequence into mRNA and introduces a premature stop codon. (C) The peptide sequence between exon 9 and 10 of the susceptible strain and truncated product of the resistant strain. Conserved bases or amino acids are shown with an asterisk (*).

Exons 2–12 of the *Pxα6* were PCR amplified with gene specific primers using cDNA generated from total RNA of 4^th^ instar spinosad susceptible (Geneva 88) or spinosad resistant (a backcross bioassay survivor) larvae. Products were excised from agarose gels (≈1500 bp), purified and reamplified with a nested reverse primer, (also within exon 12) and cloned. All 9 clones sequenced from Geneva88 (plus single clone sequenced from exons 1–12) contained the full complement of exons, and all 10 clones from BCS3-Pearl contained in addition, a frame-shifting 40 bp insertion between exons 9 and 10 creating a premature stop codon in resistant larvae (GenBank GU060294–GU060298). Genomic DNA of the BCS3-Pearl grandfather, used to generate the resistance-mapping crosses, was PCR amplified across the *Pxα6* 40 bp insertion, cloned and sequenced (GenBank GU060290). Intron 9 was approximately 6 kb shorter (1515bp in BCS3-Pearl compared to 7748 bp in Geneva88), and contained a point mutation at the 5′ donor site (GT changed to AT). Comparison with the BCS3-Pearl cDNA sequence indicated that intron splicing occurred after 40bp, at a second “GT” splice-site, not found in Geneva88 (Figure 4). This mutation has marked effects on the protein sequence and predicted transmembrane topology of the Pxα6 subunit. Although leaving the third transmembrane segment TM3 intact, it removes the 148-aa cytoplasmic loop and the 19-aa TM4 and short extracellular carboxy-terminus. No functional variants of nAChR subunits lacking the cytoplasmic loop or TM4 are known.

### nAChR Pxα6 mRNA splice variation and A-to-I mRNA editing

Considerable splice-form variation has been reported in nAChR α6 orthologues from other insect species, and this was further confirmed here for *P. xylostella*. Six out of 10 Geneva88 clones contained an additional 30 bp at the acceptor site of intron 10, which added 10 amino acids to the subunit, between TM3 and TM4. The identical 30 bp sequence was observed in BCS3-Pearl genomic DNA, but not in any of the sampled mRNA molecules. Geneva88 clones also incorporated either exon 3a or exon 3b (4 and 6 clones respectively), while all 10 BCS3-Pearl clones expressed exon 3a. Additional clone sequencing using primers positioned in the 5′ and 3′ untranslated regions confirmed the presence of exon 3b in resistant insects (GenBank GU207836). Thirty synonymous single nucleotide polymorphisms (SNPs) were identified within or between Geneva88 and the bioassay survivor (Table S1), excluding exon 3a and 3b splice variants and exon 5 A-to-I editing sites (see below). There was no clear correlation between the different splice variants described, either the additional 30 bp and exon 5 editing, the synonymous SNP variants or the alternative forms of exon 3 seen in Geneva88.

**Figure 5:**
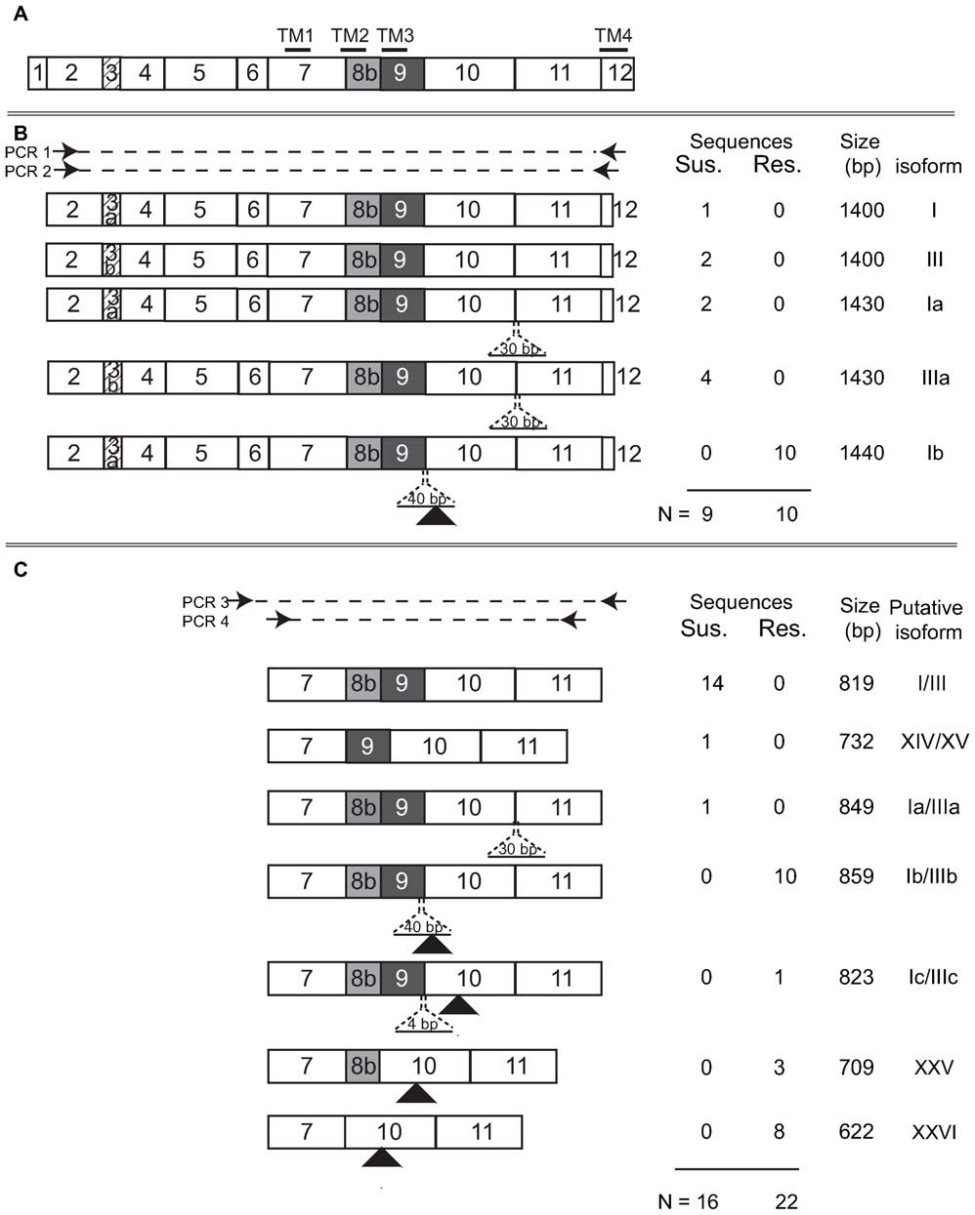
Summary of nAChR Pxα6 splice variation in resistant and susceptible *Plutella xylostella* larvae. (A) Schematic of a full-length transcript, with four transmembrane domains. Two exon 3 variants, 3a or 3b, were observed through cloning. (B) Summary of transcripts observed from PCR amplification between exons 2 and 12. PCR 1 was performed with primers Pxα6_ex2_F and Pxα6_ex12_R3, products excised from agarose gels and reamplified with nested PCR 2 using primers Pxα6_ex2_F and Pxα6_ex12_R2. Amplicon sizes are shown in base pairs (bp). Isoform names are provided in general accordance with Rinkevich and Scott [29] or new isoform numbers assigned. (C) Summary of transcripts from PCR between exons 7 and 11. PCR 3 was amplified with Pxα6_ex6F and Pxα6_ex12R, products column purified and reamplified with PCR 4, Pxα6_ex7_F and Pxα6_ex11_R. All clones sequenced from the resistant strain contained premature stop codons (black triangles). There were no stop codons or change in reading frame observed in clones from the susceptible strain. Insertions of 30, 40, or 4 base pairs are shown with dashed lines.

The observation of splicing mutations at intron 9 in the resistant strain and splicing variants of exon 11 in the susceptible strain prompted further investigation of transcripts of this specific gene region. cDNA from a resistant and a susceptible 4^th^ instar were PCR amplified using primers in exons 6 and 12, products column purified, reamplified with exon 7 and 11 primers and products cloned. Colonies were picked and amplified directly then carefully chosen for sequencing based upon amplicon size differences. In the susceptible strain, one additional splice form lacking exon 8b was detected, removing transmembrane domain 2, without a change in reading frame. Three additional splice forms were identified in the resistant strain, all of which introduced in-frame premature stop codons including i) a 4 bp insertion following the intron 9 point mutation, ii) an exon 9 exclusion and iii) exclusion of exons 8b plus 9 (Figure 5). To compare these splice variants in a broader sample set, cDNA from 4^th^ instar larvae of 12 resistant siblings from a backcross and 12 susceptible individuals were PCR amplified (as above) and products size separated using agarose gel electrophoresis. Diverse yet reproducible *Pxα6* splice patterning was observed within both resistant and susceptible larvae, however amplicon sizes differed between these groups (GenBank GU060299–GU060305, Figure S1).

A-to-I mRNA editing in exon 5 of *nAChR α6* has been reported to increase subunit diversity in many insects [28],[37]. To determine whether editing differences occur between spinosad resistant and susceptible strains, primers within exon 5 were designed for sequencing gDNA and cDNA from the same individual. Four editing sites were confirmed in both susceptible and resistant strains and, based upon the numbering system outlined in Jin *et al.* (2007), sites 5, 6 and 10 were conserved with *H. virescens*, *B. mori* and *D. melanogaster* while site 4 was in the same codon but edited a different non-synonymous base (Figure S2).

## Discussion

We used genetic mapping to identify for the first time, a locus underlying field evolved resistance to the widely used bioinsecticide spinosad, in the insect pest *Plutella xylostella*. A point mutation in the *nAChR Pxα6* gene predicted to produce a truncated subunit was discovered in spinosad resistant individuals. As this mutation originated from the field and not from a laboratory selection experiment, this finding will enable field monitoring for a relevant resistance allele in this global insect pest of brassica crops, and also aid studies of resistance to spinosad in other insects. Convergent evolution of the genetic mechanisms controlling resistance to insecticides is common across insect orders because the same essential targets are involved. For example, resistance to cyclodienes has been associated with the same amino acid substitution in the GABA gated chloride ion channel in Diptera, Coleoptera and Dictyoptera [38] while laboratory selected resistance to Bt toxins in Lepidoptera can involve various mutations in a midgut cadherin-like protein [39]–[41]. Thus, molecular characterization of the mechanism of resistance to spinosad in diamondback moth provides strong candidates to search for similar mutations across other insect genera.

Insecticides have an essential role in controlling pests in modern agriculture, and management strategies to minimize the evolution of resistance can play a critical role in maintaining productivity. Identification of specific resistance mutations can enable screening assays to be developed for early monitoring of the spread of resistance alleles. This is particularly important for genetically recessive resistance alleles, such as that studied here, where the phenotypic expression of resistance is very rare when the alleles first arrive in a population. *P. xylostella* populations have typically developed resistance rapidly after sustained application of spinosad [7]. Crosses testing allelic complementation in field evolved resistant populations of *P. xylostella* have shown that allelic mutations in the same resistance gene are present in three US states, Hawaii, Georgia and California [32]. When any two genetically recessive spinosad resistant populations were crossed, F_1_ progeny were resistant, demonstrating a shared resistance gene. These crossing results and our molecular findings predict that mutations in the nAChR *Pxα6* cause spinosad resistance in all these field evolved populations, however it remains to be seen whether the same intron 9 point mutation is present in every case. Genetic assays for monitoring the presence of resistance alleles, even in untreated populations, can be developed at the *Pxα6* locus isolated here, similar to population screening approaches employed for cadherin mutations in Bt resistance [42],[43].

### The spinosad target site

Several classes of insecticide target nAChR's including neonicotinoids and spinosad. Recently spinosyn A, the primary component of spinosad, was shown to act independently of known binding sites on nAChRs for other compounds, including the site for the neonicotinoid insecticide, imidacloprid [44]. Orr and colleagues conclude that a novel mode of action is responsible for spinosad toxicity that does not involve known ligand binding domains. The truncation of the *Pxα6* coding sequence after exon 9 in the mutant may indicate that spinosad is interacting with the wild type nAChR molecule at the intracellular receptor loop between TM3 and TM4, which is removed by this truncation. These loops are thought to be involved with receptor biosynthesis and assembly, and can affects the rate at which current flows through the receptor's channel [45]. Alternatively, spinosad may interact with the extracellular carboxy-terminus of the protein, although this seems unlikely as only 8 amino acids are predicted outside the membrane. Both regions are also deleted in the *Drosophila* spinosad-resistant CyO allele of *Dα6*, as well as TM3, due to the occurrence of one of the CyO inversion breakpoints within exon 8b. Thus any protein expressed by this *Drosophila* strain would lack the TM3 and downstream domains. Alternatively, transcripts with truncated CDS may produce entirely non-functional proteins, or the transcripts may be degraded through non-sense mediated decay. Whatever the exact mechanism, the high levels of resistance conferred by both the resistance mutation identified here in *Plutella*, and the truncation mutation previously identified in *Drosophila*, indicates that the nAChR *α6* subunit is the prime target of spinosad action.

### Post transcriptional modification and splicing

Insect nAChR genes can exhibit extensive splice-form variation and other post-transcriptional modification. Notably, frameshifts caused by alternate exon splicing or incorrect intron splicing have been reported in nAChRs from *T. castaneum*, *A. mellifera* and *D. melanogaster* and *Anopheles gambiae* [27]–[29]. It is unclear whether these shortened fragments have a functional role, however they are likely to have a profound effect on channel properties [25]. It has been suggested that alternatively spliced products of nAChR genes may act as acetylcholine “sponges”, or influence expression of full-length transcripts [25],[28]. The presence of truncated protein molecules in wild-type genetic backgrounds may suggest that these are only mildly deleterious, and perhaps might indicate that the recessive resistance allele could have been present even before the advent of spinosad insecticides. This may explain the rapid appearance of resistance in *Plutella*. To search for additional mis-splice mutations, *Pxα6* exons 7 to 11 were amplified. Multiple frameshift mutations were identified in a resistant larva due to the presence of the intron 9 point mutation or complete exclusion of the mutation containing exon. In contrast, all transcripts sequenced from susceptible larvae maintained the correct translational reading frame.

It is interesting to note, that in the housefly, sequence variation in subunit *Md*α6 did not show an association with laboratory generated spinosad resistance. Nonetheless, a single *Mdα6* clone showed a similar frameshift mutation, due to incorrect splicing of intron 9, a mutation in the same gene region as shown here in *Pxα6* [31]. Whether this region of the gene is prone to mutations remains unclear, however, we speculate that similar resistance mechanisms as those described in *Plutella* could arise in other insects experiencing similar selective pressures.

Although there may be a fitness cost associated with resistance [46], full length transcripts of the α6 gene are apparently not necessary for survival. High levels of protein sequence identity across insect orders would seem to indicate strong stabilising selection on protein function. However, spinosad resistant strains of *Plutella xylostella* have survived under laboratory conditions for more than 7 years, although costs of resistance may not be fully expressed in laboratory conditions. Whatever is the case, knockout or truncation mutations are not particularly common causes of field evolved insecticide resistance, presumably as insecticide target molecules are generally, almost by definition, functionally important and therefore knockout mutations in target molecules will tend to be lethal. However, the existence of several genes encoding nAchR α-type subunits may allow for some functional redundancy, if another subunit can be recruited to substitute for a defective Pxα6 protein. It will clearly be interesting to further investigate how and when this truncation mutation in *Pxα6* arose, its molecular mode of action in conferring resistance, and to identify any associated fitness costs. Identification of the molecular changes in the *Pxα6* gene associated with resistance is a key step towards all of these goals.

## Materials and Methods

### Insects and crosses

The spinosad susceptible strain of *P. xylostella*, Geneva 88, was collected from Geneva, NY in 1988 and maintained on artificial diet without insecticide exposure. The spinosad resistant strain Pearl-Sel was collected from Oahu, Hawaii in 2001 and was 1080 fold resistant to spinosad at generation F5 [5]. Selection of Pearl-Sel with spinosad under laboratory conditions increased the toxicity ratio to 18,600 fold. Pearl-Sel was crossed to Geneva 88 for two generations, selected for survival on artificial diet for laboratory rearing, then backcrossed to Geneva 88 for three times and re-selected for spinosad resistance, resulting in BCS3-Pearl used in this study. Spinosad bioassays were prepared by soaking artificial diet in liquid spinosad (SpinTor 2 SC) for two hours, excess fluid drained, and residual droplets air dried. Second instar larvae were used in bioassays and reared on diet containing insecticide until pupation.

Prior to mapping crosses, BCS3-Pearl larvae were treated with a diagnostic dose (10 ppm) of spinosad. Single pair matings were established between a BCS3-Pearl male and Geneva 88 female. Some F_1_ individuals were bio-assayed to confirm that resistance was recessive. Single pair backcrosses were then established between a BCS3-Pearl male and F_1_ female. Some backcross progeny were reared to adult then 32 untreated controls frozen (−80°C) while ∼70 of the progeny were treated with a diagnostic dose of spinosad and 35 survivors frozen. A second series of crosses were established for male informative crosses for recombinational mapping. Male informative mapping families were generated from 31 F_1_ brothers who were backcrossed to BCS3-Pearl females in single pair matings. Bioassays were performed using 15 ppm spinosad, and produced 2315 survivors that were related by a single grandparental cross.

### Nucleic acid preparation and analysis

Genomic DNA extraction procedures were performed according to Zraket *et al.* (1990) [47]. Total larval RNA was extracted using RNeasy kit (Qiagen). Reverse transcription of total RNA was performed with BioScript (Bioline) using a random hexamer (0.2 µg).

AFLPs were performed on 100–200 ng of genomic DNA according to Vos *et al.* (1995) using 11 primer combinations with three selective bases (EcoANN-MseCNN) [48]. AFLP Eco primers were labelled with γ-^32^P or γ-^33^P and separated on 6% polyacrylamide gels and exposed on X-OMAT film (Kodak) for 1 to 7 days depending on the strength of the isotope. AFLP bands were analysed manually. MapMaker v2.0 was used to assemble raw AFLP data into linkage groups function with LOD ≥3.00 and θ≤0.40, using both genotype phases.

Specific primers were designed using Oligo 6.4 (Molecular Biology Insights) or Primer3 [49] (Table S2). PCR reaction volumes were between 10µl and 50µl using Taq polymerase (Bioline) with final reaction concentrations: buffer (1×), MgCl_2_ (2 mM), dNTP (0.1 mM), primer (0.2 mM), Taq polymerase (0.5 units). Extensor enzyme (Thermo Scientific) was used for genomic DNA and cDNA clone amplification. Template concentrations ranged from 3ng–100ng of genomic DNA and 1–2 µl of cDNA template generated from reverse transcription reactions. Clones were obtained by ligating PCR products into pGEM T-easy vector system (Promega, WI, USA) or CopyControl (cambio). DNA sequencing reactions were prepared using Big Dye 3.1 and sequenced using a 3730×l Capillary Sequencer (ABI). Sequence analysis was performed using CodonCode Aligner. Multiple cDNA clones were sequenced from single individuals to distinguished polymorphic sites from cloning errors. The sequences reported in this paper have been deposited in the GenBank database (GU058050, GU207835, GU207836, GU060290–GU060305).

### Gene mapping and genotyping

Degenerate primers were designed by aligning nAChR α6 protein sequences with MacVector 7.0 (Accelrys) [*H. virescens* (AAD32698), *D. melanogaster* (Q86MN8), *B. mori* (ABV45518), *A. gambiae* (XP_308042)]. Genotyping was performed using PCR amplification and agarose gel electrophoresis for a female informative cross with *PxDα6* primers Pxα6_ex7_F×Pxα6_ex8_R. In male informative crosses, Pxα6_Intron5F×Pxα6_Intron5R was digested with *BsrG1* (NEB) and Pxα6_ex11_F×Pxα6_ex12_R digested with *AluI* (NEB). The location of *nAChR a6* was identified in the genome of *Bombyx mori* (silkdb, nscaf2838) and flanking genes were BLAST against *P. xylostella* 454-ESTs to obtain gene specific sequence. *PPTSR* (GenBank GU060291) was amplified with PPTSR_F, PPTSR_R and digested *MscI* (NEB) and *arginine kinase* (GenBank GU060292) using ArgKin_F×ArgKin_R, digested with *Taq alpha1* (NEB).

### 454-EST library construction and sequencing

Messenger RNA was purified from Geneva 88 eggs and all larval stages using TRIzol reagent (Invitrogen) and larval midguts by the RNeasy MinElute Clean up Kit (Qiagen). Genomic DNA was removed by incubation with DNAse (TURBO DNAse, Ambion) for 30 min at 37°C. RNA integrity and quantity was verified on an Agilent 2100 Bioanalyzer using the RNA Nano chips (Agilent Technologies) and Nanodrop ND-1000 spectrophotometer. Full-length enriched, normalized cDNAs were generated from 2 µg of total RNA using the Creator SMART cDNA library construction kit (BD Clontech). Reverse transcription was performed with a mixture of several reverse transcription enzymes for 60 min at 42°C and 90 min at 50°C. Double-stranded cDNAs were normalized using the trimmer-direct cDNA normalization kit (Evrogen) to reduce abundant and increase rare transcripts. This normalized larval cDNA was used as a template for 454-FLX sequencing which resulted in a total of 68.9 Mb from 315367 reads, clustered into 19,309 contigs using Newbler software (Liverpool, UK).

### BAC library and screening and genomic sequencing

A *P. xylostella* genomic BAC library was constructed using Geneva 88 after partial digestion with restriction endonuclease *Mbo*I and ligating into vector pIndigoBAC536 (Clemson University Genomics Institute). The average insert size was 109.4 kb which provided 7.6× genome coverage from 23,808 clones. A *nAChR Pxα6* sequence amplified from cDNA (primers Pxα6_ex7_F×Pxα6_ex11_R) was ^33^P labelled using Prime-a-Gene labelling kit (Promega) and used to screen the library. Five clones were identified (Px7p6, Px8d14, Px10h8, Px14d18, Px17d20, where Px = *Plutella xylostella*, followed by plate number and grid position) and Px8d14 selected for sequencing (GenBank GU058050). Clone annotation was performed using the *B. mori* annotation program KAIKOGAAS (http://kaikogaas.dna.affrc.go.jp/) and BLASTn searching against *P. xylostella* 454-ESTs. The BCS3-Pearl grandfather used to produce all male informative mapping families was PCR amplified with primers Pxα6_ex9_F×Pxα6_ex10_R and Pxα6_ex10_F×Pxα6_ex12_R and assembled into a single sequence (GenBank GU060290).

### nAChR Pxα6 cDNA amplification

PCR primers predicted to be within *nAChR Pxα6* 5′ and 3′ untranslated mRNA regions (Pxα6_5prime_F1×Pxα6_3primeR1) were used to amplify a product from Geneva 88 with Extensor polymerase (GenBank GU207835). SignalP 3.0 predicted the signal peptide cleavage site [50], transmembrane domains predicted with TMpred program (http://www.ch.embnet.org/software/TMPRED_form.html) and ProSite identified the neurotransmitter gated ion-channels signature [51] (Figure 3).

A single 4th instar backcross (R(RxS)) larvae that survived a spinosad bioassay and a single Geneva 88 4^th^ instar larva were amplified with primers in exon 2 (Pxα6_ex2_F) and 12 (Pxα6_ex12_R3). Products were excised from 1.5% agarose gel and re-amplified with the same forward primer and slightly nested reverse primer, also in exon 12 (Pxα6_ex12_R2). dATP overhangs were added and products cloned into pGEM-t-Easy vector. Nine clones from G88 and 10 clones from BCS3-Pearl were amplified with proof-reading taq polymerase and sequenced with vector primers (T7 and SP6) plus one internal primer located within exon 6 (Pxα6_ex6_F) (GenBank GU060294–GU060298). nAChR *Pxα6* was amplified from cDNA of multiple Geneva 88 and BCS3-Pearl larvae with exon 6 and 12 primers (Pxα6_ex6_F×Pxα6_ex12_R), products were purified using MinElute columns (Qiagen) then reamplified using exon 7 and 11 primers (Pxα6_ex7_F×Pxα6_ex11_R). One individual from each strain was cloned and sequenced (GenBank GU060299–GU060305), and remainder run on agarsoe gel (1.5%, 12 hour 50 volts).

## Supporting Information

Figure S1 An example of *Pxα6* transcript diversity in *Plutella xylostella* 4^th^ instar larvae. Total RNA was isolated from 4^th^ instar larvae of 12 susceptible individuals and 12 spinosad resistant siblings from a backcross. cDNA was generated and amplified using primers Pxα6_ex6_F and Pxα6_ex12_R, products column purified and reamplified with Pxα6_ex7_F and Pxα6_ex11_R. Similar splicing patterns are observed within susceptible or resistant individuals, but not shared between the two groups. Expression of splice variants may alter rapidly during development. Prominent BCS3-Pearl PCR bands are i) 859/823 bp (exons 7,8b,9, 10,11 plus 40 or 4 bp insertion between exons 9 and 10), ii) 709 bp (exons 7,8b,10,11), iii) 622 bp (exons 7,10,11). Additional bands are present. Prominent PCR band sizes in Geneva 88 are iv) 819 bp (exons 7,8b,9,10,11) and 849 bp (exons 7, 8b, 9, 10, 11, +30 bp insert between 10 and 11), v) 732 bp (7,9,10,11). Additional bands are present.
(2.26 MB EPS)

Figure S2 Comparison of lepidopteran A-to-I mRNA editing sites in nAChR α6, exon 5. (A) Adenine to guanine editing sites are shaded and numbered according to Jin *et al.* (2007) [37]. *Heliothis virescens* (Hv) and *Bombyx mori* (Bm) share the same editing sites, however *Plutella xylostella* (Px) differs at site 4 (4b in Px). The possible amino acids encoded by the adenine (A) or guanine (G) are shown. Dots indicate common bases or amino acids. (B) Ten clones were sequenced from a spinosad susceptible or resistant 4^th^ instar larva. Exon 5 edited amino acids haplotypes for sites 4(b), 5 and 6, and 10 are shown. Based on predicted coding sequence of nAChR *Pxα6*, (GU058050) sites 4b, 5, 6, and 10 correspond to bases 391, 394, 395, and 447 respectively and amino acids 131, 132, and 149.
(0.49 MB EPS)

Table S1 Polymorphic sites identified within and between *Plutella xylostella* strains.
(0.08 MB DOC)

Table S2 PCR primer sequences.
(0.08 MB DOC)
