# Supplementary material for: Mis-Spliced Transcripts of Nicotinic Acetylcholine Receptor α6 Are Associated with Field Evolved Spinosad Resistance in Plutella xylostella (L.)
Source: PLoS Genet. 2010 Jan 8;6(1):e1000802. doi: 10.1371/journal.pgen.1000802 (PMC2792709; doi:10.1371/journal.pgen.1000802)
Supplement: Table S1 — Polymorphic sites identified within and between Plutella xylostella strains. (0.08 MB DOC) [file pgen.1000802.s003.doc]

| Base position^1^ | Px8d14 (Geneva 88) | Geneva 88 | Resistant | Amino Acid |
| --- | --- | --- | --- | --- |
| 88 | T | T | C | L |
| 99 | G | G | A | S |
| 657 | C | C | T | D |
| 729 | C | C/G | C | L |
| 768 | G | G/C | G | P |
| 915 | G | G/C | G | A |
| 928 | C | C/T | C | L |
| 999 | A | A | G | S |
| 1008 | G | G/T | G | L |
| 1020 | C | C | G | P |
| 1026 | A | A | C | I |
| 1038 | A | A | T | S |
| 1059 | C | C/G | C | T |
| 1173 | G | G | T | P |
| 1188 | T | C | T | S |
| 1191 | C | C/T | C | T |
| 1197 | T | T/C | T | S |
| 1200 | T | T/G | T | T |
| 1248 | T | T/C | T | R |
| 1251 | C | C/G | C | S |
| 1281 | G | G/C | C | V |
| 1302 | C | C | T | L |
| 1317 | T | T | C | H |
| 1320 | C | C | G | R |
| 1338 | G | G | A | L |
| 1341 | G | G | A | R |
| 1383 | G | G/A | G | E |
| 1413 | T | T/C | T | F |
| 1416 | T | T/C | C | A |
| 1425 | C | C/T | T | V |

Table S2. Polymorphic sites identified within and between *Plutella xylostella* strains

^1^Numbering based upon BAC 8d14 predicted coding sequence of nAChR *Pxα6*, GU058050. Variable exons 3a, 3b and A-to-I mRNA editing sites not shown.
