# Supplementary material for: Mis-Spliced Transcripts of Nicotinic Acetylcholine Receptor α6 Are Associated with Field Evolved Spinosad Resistance in Plutella xylostella (L.)
Source: PLoS Genet. 2010 Jan 8;6(1):e1000802. doi: 10.1371/journal.pgen.1000802 (PMC2792709; doi:10.1371/journal.pgen.1000802)
Supplement: Table S2 — PCR primer sequences. (0.08 MB DOC) [file pgen.1000802.s004.doc]

Table S1. PCR primer sequences

| **Gene Mapping and Genomic DNA Amplification** | | |
| --- | --- | --- |
| Pxα6_ex7_F | GGGCAAGAAGAACACGATAAC | Gene mapping in female informative backcross |
| Pxα6_ex8_R | GCCACCAAATTGAGGAACAC |
| Pxα6_Intron5F | TTTTGCATGGAGCAAAAGAA | Gene mapping in male informative backcross (within intron 5) |
| Pxα6_Intron5R | GGAGTCATGCAAGGAGTGGT |
| Pxα6_ex11_F | GAYTGGAARTTYGCNGCNATGGT | Gene mapping in male informative backcross (across intron 11) |
| Pxα6_ex12_R | GCNACNGTNGCDATDATNGTRAA |
| PPTSR_F | TGAAAACATAGTTGGCCCTGA | Mapping of  *phosphatidylserine receptor* |
| PPTSR_R | ATCTCGTTGCTTGCCTCCTA |
| ArgKin_F | GATGTTGAGACCCTCGGAAA | Mapping of *arginine kinase* |
| ArgKin_R | GATGCCGTCGTACATCTCCT |
| Pxα6_ex9_F | TGGTGCTCAACTACCACCAC | Amplification of BCS3-Pearl genomic DNA |
| Pxα6_ex10_R | CCAGCAGAGACTTGGAGGAC |
| Pxα6_ex10_F | GATACTGCGCATGTCACGTC | Amplification of BCS3-Pearl genomic DNA |
| Pxα6_ex12_R | TGAACACGAACAGGCAAAAC |
| **cDNA amplification** | | |
| Pxα6_ex7_F | GGGCAAGAAGAACACGATAAC | Amplification of Pxα6 from Geneva 88 cDNA. This product was used to screen the *P. xylostella* BAC library |
| Pxα6_ex11_R | ACCATNGCNGCRAAYTTCCARTC |
| Pxα6-deg_ex4_F | TGGAAGCCCGAYGTNCTNATGTA | Degenerate PCR amplification used to identify the Pxα6 gene |
| Pxα6-deg_ex9_R | ACCATRAACATDATRCARTTRAA |
| Exon5_RNAeditF | GAAGGTTTTGACGGGACGTA | Pxα6 exon 5 amplification using genomic DNA and cDNA from the same individual, to detect A-to-I mRNA editing. |
| Exon5_RNAeditR | GGTCCAGCTACCGAACTTCA |
| Pxα6_5prime_F1 | GTGCGCGGGTGTGGTATG | Amplification of full length coding sequence (Figure 3, GU207835) |
| Pxα5_3prime_R1 | GTTCTCTCGTGAGGGCAATG |
| Pxα6_ex2_F | ACGAGAAGCGTTTGCTGAAC | Pxα6 PCR 1 and 2 (see Figure 5B) |
| Pxα6_ex12_R3 | CGCGATGATCGTGAACAG |
| Pxα6_ex12_R2 | CGTGAACAGCGTGAACACGAA |
| Pxα6_ex6_F | TTCATCACCAACGGAGAATG | Pxα6 PCR 3 (see Figure 5C) |
| Pxα6_ex12_R | TGAACACGAACAGGCAAAAC |
| Pxα6_ex7_F | GGGCAAGAAGAACACGATAAC | Pxα6 PCR 4 (see Figure 5C) |
| Pxα6_ex11_R | ACCATNGCNGCRAAYTTCCARTC |
